# Seizure control through genetic and pharmacological manipulation of Pumilio in *Drosophila*: a key component of neuronal homeostasis

**DOI:** 10.1242/dmm.027045

**Published:** 2017-02-01

**Authors:** Wei-Hsiang Lin, Carlo N. G. Giachello, Richard A. Baines

**Affiliations:** Division of Neuroscience and Experimental Psychology, School of Biological Sciences, Faculty of Biology, Medicine and Health, University of Manchester, Manchester M13 9PT, UK

**Keywords:** Anticonvulsant, *Drosophila*, Epilepsy, Pumilio, Sodium current, Translational repression

## Abstract

Epilepsy is a significant disorder for which approximately one-third of patients do not respond to drug treatments. Next-generation drugs, which interact with novel targets, are required to provide a better clinical outcome for these individuals. To identify potential novel targets for antiepileptic drug (AED) design, we used RNA sequencing to identify changes in gene transcription in two seizure models of the fruit fly *Drosophila melanogaster*. The first model compared gene transcription between wild type (WT) and *bangsenseless^1^* (*para^bss^*), a gain-of-function mutant in the sole fly voltage-gated sodium channel (*paralytic*). The second model compared WT with WT fed the proconvulsant picrotoxin (PTX). We identified 743 genes (FDR≤1%) with significant altered expression levels that are common to both seizure models. Of these, 339 are consistently upregulated and 397 downregulated. We identify *pumilio* (*pum*) to be downregulated in both seizure models. Pum is a known homeostatic regulator of action potential firing in both flies and mammals, achieving control of neuronal firing through binding to, and regulating translation of, the mRNA transcripts of voltage-gated sodium channels (Na_v_). We show that maintaining expression of *pum* in the CNS of *para^bss^* flies is potently anticonvulsive, whereas its reduction through RNAi-mediated knockdown is proconvulsive. Using a cell-based luciferase reporter screen, we screened a repurposed chemical library and identified 12 compounds sufficient to increase activity of *pum*. Of these compounds, we focus on avobenzone, which significantly rescues seizure behaviour in *para^bss^* flies. The mode of action of avobenzone includes potentiation of *pum* expression and mirrors the ability of this homeostatic regulator to reduce the persistent voltage-gated Na^+^ current (I_NaP_) in an identified neuron. This study reports a novel approach to suppress seizure and highlights the mechanisms of neuronal homeostasis as potential targets for next-generation AEDs.

## Abstract (precis)

**Summary:** Next-generation anticonvulsant compounds potentiate the activity of the neuronal homeostatic regulator Pumilio.

## INTRODUCTION

The number of known contributory genetic loci to human seizure exceeds 500, which greatly increases the challenge of providing personalised medicine through tailoring treatments based on individual gene mutation (Noebels, 2015). An alternative is to target treatment to common modifiers to which larger groupings of individual gene mutations contribute. One obvious modifier is neuronal homeostasis, which acts to stabilise neural circuit activity levels through continual adjustment of neuron excitability (Turrigiano, 2012). However, this opportunity remains unexplored.

Seizures in humans and *Drosophila* exhibit sufficient parallels to implicate that the underlying neuronal abnormalities are highly similar. This includes defined seizure thresholds, common genetic mutations that modify seizure susceptibility, spread of seizures along defined neuronal tracts and suppression of seizures by recognised AEDs (Muraro and Baines, 2008; Song and Tanouye, 2008). As in humans, certain mutations in *Drosophila* genes result in a seizure phenotype (collectively termed bang-sensitive). Seizures can also be induced in *Drosophila* by exposure to proconvulsants, including picrotoxin (PTX), primarily through block of inhibitory GABA_A_ receptors (Lin et al., 2012).

To model seizure, we used *para^bss^*, which is a L1699F point mutation that imparts a gain of function in the sole voltage-gated sodium channel (Na_V_) of the fly genome (Parker et al., 2011). Mutations in the human ortholog, *SCN1A*, are associated with seizure and intractable epilepsy (Escayg and Goldin, 2010). In comparison, we also used exposure to PTX. We exploited the molecular tractability of *Drosophila* to identify changes to gene transcription that occur during seizure to identify possible pathway nodes exploitable for anticonvulsant therapy. Comparison between the two models identifies 743 common transcriptional changes, including *pum*. Pum is a translational repressor that binds mRNA transcripts that normally (but not exclusively) contain an 8-nucleotide binding motif in their 3′-UTR, termed a Nanos response element [NRE, also known as Pumilio response element (PRE)] (Gerber et al., 2006). A particularly relevant Pum target, with respect to seizure, is Na_v_. We have previously shown that the fly Na_v_ (*paralytic*) is translationally regulated by Pum and also that rat *Scn8a* (*Na_v_1.6*) is similarly regulated by *Pum2* (the closest mammalian homologue to *pum*) (Driscoll et al., 2013; Mee et al., 2004; Muraro et al., 2008). This mechanism forms part of a well-characterised homeostatic response that tunes action potential firing to match the changing level of synaptic excitation to which neurons are exposed (Baines, 2005; Weston and Baines, 2007). Two recent studies highlight the potential involvement of Pum in epilepsy. First, a *Pum*2 knockout mouse exhibits spontaneous seizures (Siemen et al., 2011) and second, *PUM*2 expression is reduced in human patients suffering temporal lobe epilepsy (TLE) (Wu et al., 2015).

We show here that overexpression of *pum* in *para^bss^* flies is markedly anticonvulsant. By contrast, RNAi-mediated knockdown of *pum* exacerbates seizure. The likely beneficial effect of upregulation of Pum is through reduction of the voltage-gated persistent sodium current (I_NaP_) in central neurons. Thus, our results highlight mammalian Pum2 as a potential target for the design of novel, and possibly wide-spectrum, AEDs. To identify potential compounds that influence Pum activity and/or expression, we constructed a luciferase reporter of Pum activity and screened a comprehensive library of approved compounds. From 785 compounds, we identify 12 that potentiate Pum activity. Further analysis of one of these compounds, avobenzone, shows that it increases transcription of *pum*, reduces I_NaP_ in identified motoneurons and is potently anticonvulsive in *Drosophila*.

## RESULTS

### RNA-sequencing identifies *pum* as downregulated in seizure

**Fig. 1.**
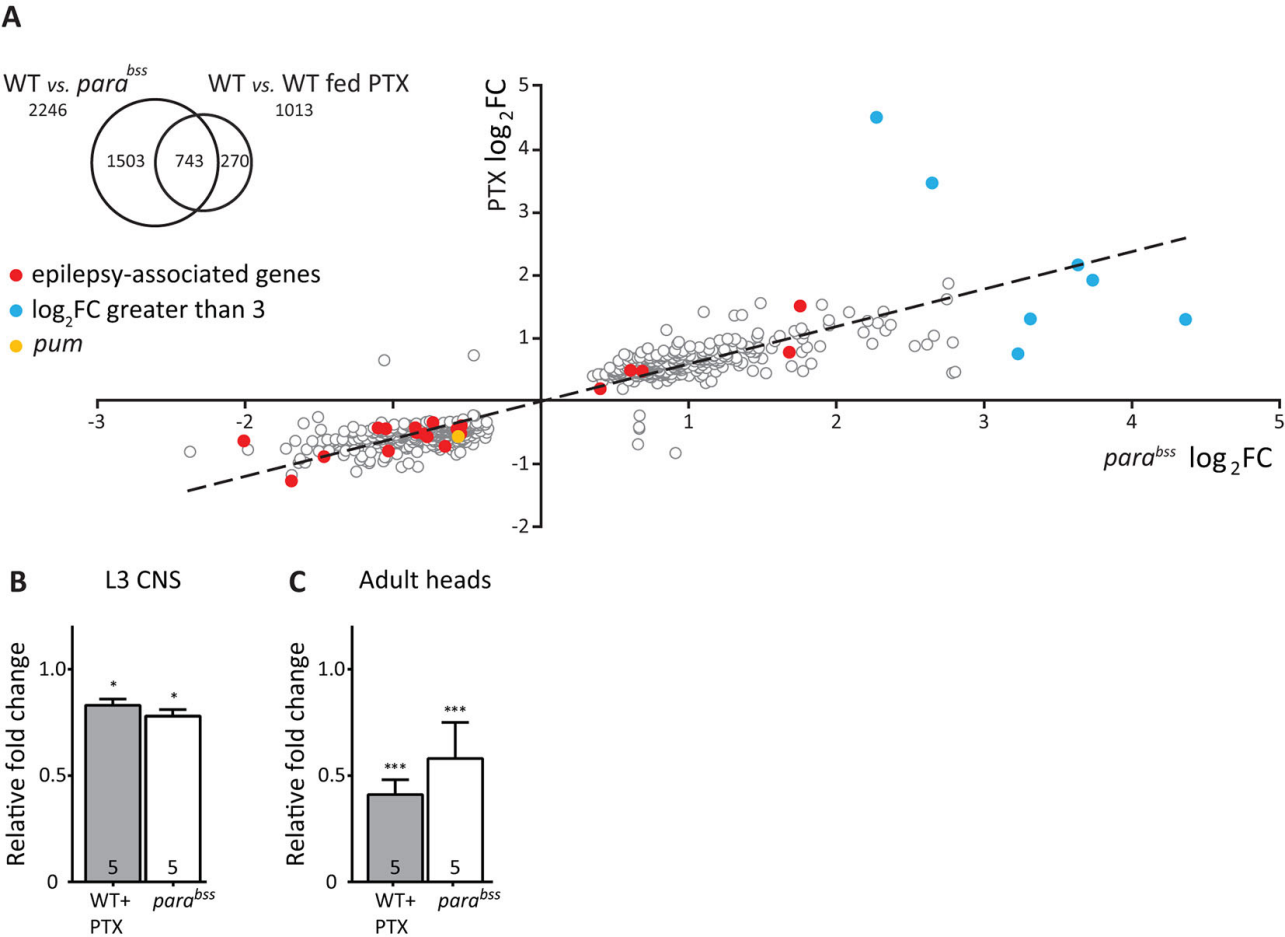
**Analysis of altered gene transcription in seizure models.** (A) Cross-comparison shows 743 changes are common to both seizure models. Analysis of direction of log_2_ fold-change (log_2_FC) in transcription of the 743 common genes (main figure) shows that 339 are significantly (two-way ANOVA) upregulated and 397 downregulated. Seven genes show differential expression in the two models. *pumilio* (*pum*), which is downregulated, is identified by the orange dot. Genes previously linked to human epilepsy are shown by red dots (described in Table 1). Blue dots highlight genes that show particularly large fold-changes (log_2_FC>3) in expression levels in the seizure backgrounds (see Results text for identity). Inset: analysis of the transcriptome by RNA-sequencing shows change to transcription of 2246 genes in the *para^bss^* CNS compared with wild type (WT). Comparison of WT with WT fed picrotoxin (PTX) shows 1013 transcriptional changes. (B) Analysis of *pum* transcript level in isolated CNS from L3 shows a significant reduction in WT+PTX and *para^bss^* compared with WT controls. (C) *pum* is significantly reduced in adult heads in both seizure models compared with WT controls. The WT value has been set to 1 in each experimental condition. Data are mean±s.d. for *n*=5 independent samples, *P≤0.05. ***P≤0.001 (unpaired *t*-test).

In order to determine changes to gene transcription that occur in seizure-prone CNSs, we used RNA sequencing (RNA-seq) to compare gene transcription in the CNS in two models of seizure: a genetic model (*para^bss^*) and a chemical model (PTX). Using RNA extracted from the CNS of third instar larvae (L3), we identified transcriptional change in 2246 and 1013 genes, respectively, using an FDR≤1% in WT versus *para^bss^* and WT versus WT fed PTX (see Tables S1, S2). Comparison between data sets revealed that 743 common genes exhibit significant change to expression (Fig. 1A inset, see Table S3 for gene details). Of these, 736 showed significant and consistent altered expression in both seizure models. A log_2_ plot of fold-change (log_2_FC) showed that 339 (46%) are significantly upregulated and 397 (54%) are significantly downregulated (*P*=0.001, ANOVA). The remaining seven genes did not show consistent direction of change (Fig. 1A). Identified genes generated a total of 130 functional clusters representing a wide array of functions, including predicted genes encoding ion channels and synaptic proteins (detailed below). The top 20 enriched clusters are shown in Fig. S1. The top four clusters are for genes associated with pre-replicative complex assembly, eukaryotic translation elongation factor 1 complex, negative regulation of neuroblast proliferation and translation repressor activity. Genes associated with translational repression include *minichromosome maintenance* (orthologues *2*, *3*, *5*, *7*), *elongation factor 1α100E*, *1α48D* and *1β*, *anachronism*, *prospero*, *musashi*, *embryonic lethal abnormal vision*, *brain tumor* and *pum* (Table S3). Twenty genes that we identify have been positively associated with human epilepsy (http://www.informatics.jax.org/humanDisease.shtml) (red dots in Fig. 1A, and described in Table 1). Of these genes, five were upregulated and 15 were downregulated in the *Drosophila* seizure models. These genes include *paralytic* (Na_v_), *nicotinic Acetylcholine* *Receptor α5*, *I_h_* *channel* and *Shaker* (K^+^ channels) in addition to *Syntaxin*, *Synapsin* and *unc-13* (synaptic proteins). Seven genes were identified that show particularly large increases in transcription (>3 log_2_FC, blue dots in Fig. 1A). These genes are CG18331 (*mucin 68Ca*), CG34076 (*mitochondrial NADH-ubiquione oxidoreducatse chain 3*), CG11205 (*photorepair*), CR41620/CR40734 (rRNA genes) and CG7606/CG32198 (unknowns).

**Table 1.**
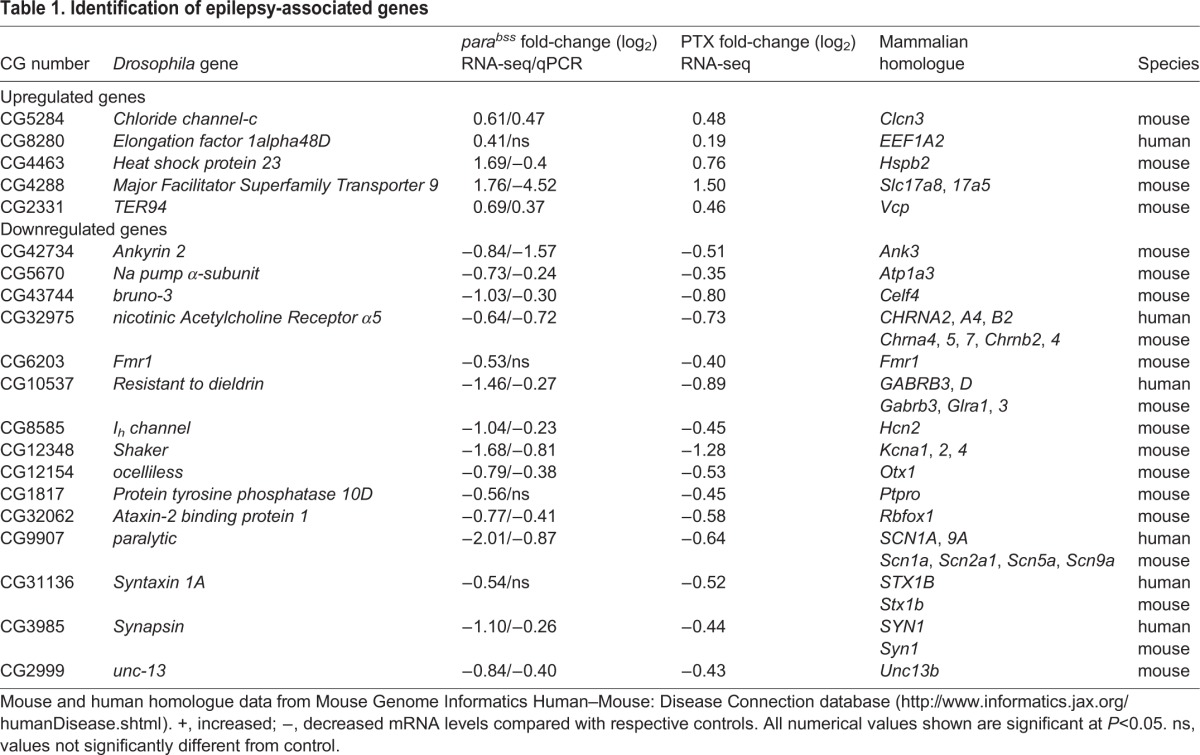
**Identification of epilepsy-associated genes**

Our attention was drawn to *pum*, which was significantly downregulated in both seizure models [mean±s.d., WT: 602±14 vs WT fed PTX: 405±2 and *para^bss^*: 381±15 cpm (counts per million), *P*=1.5×10^−5^, *n*=3]. This is because its homologue, *PUM2*, has been reported to be downregulated in humans suffering temporal lobe epilepsy (Wu et al., 2015). Pum is a well­-characterised translational repressor, which we have previously reported regulates translation of Na_v_s in both *Drosophila* and rat to achieve homeostatic control of neuron action potential firing (Driscoll et al., 2013; Mee et al., 2004; Muraro et al., 2008). We considered that manipulation of a homeostatic regulator might represent a promising approach to control seizure. To validate RNA-seq data, we undertook RT-qPCR; *pum* was significantly decreased in WT larval CNS after exposure to PTX (0.83±0.03) and in *para^bss^* (0.78±0.03) compared with WT control (set as 1, *P*=0.03, *n*=5; Fig. 1B). We observed a similar and significant downregulation of *pum* transcription in adult heads that contain mostly brain tissue (WT fed PTX: 0.41±0.07 and *para^bss^*: 0.58±0.17) relative to WT control (set as 1, *P*=0.0002, *n*=5; Fig. 1C). In addition, we used RT-qPCR to validate the identification of the 20 genes that have been positively associated with epilepsy (red dots in Fig. 1A). This validation was undertaken only for the *para^bss^* background. We found consistent change for 14 of the genes (representing a validation rate of 70%). Two genes showed significant change by RT-qPCR but in the opposite direction to RNA-seq, whereas four genes showed no significant change (see Table 1).

### Upregulation of Pum is anticonvulsive

**Fig. 2.**
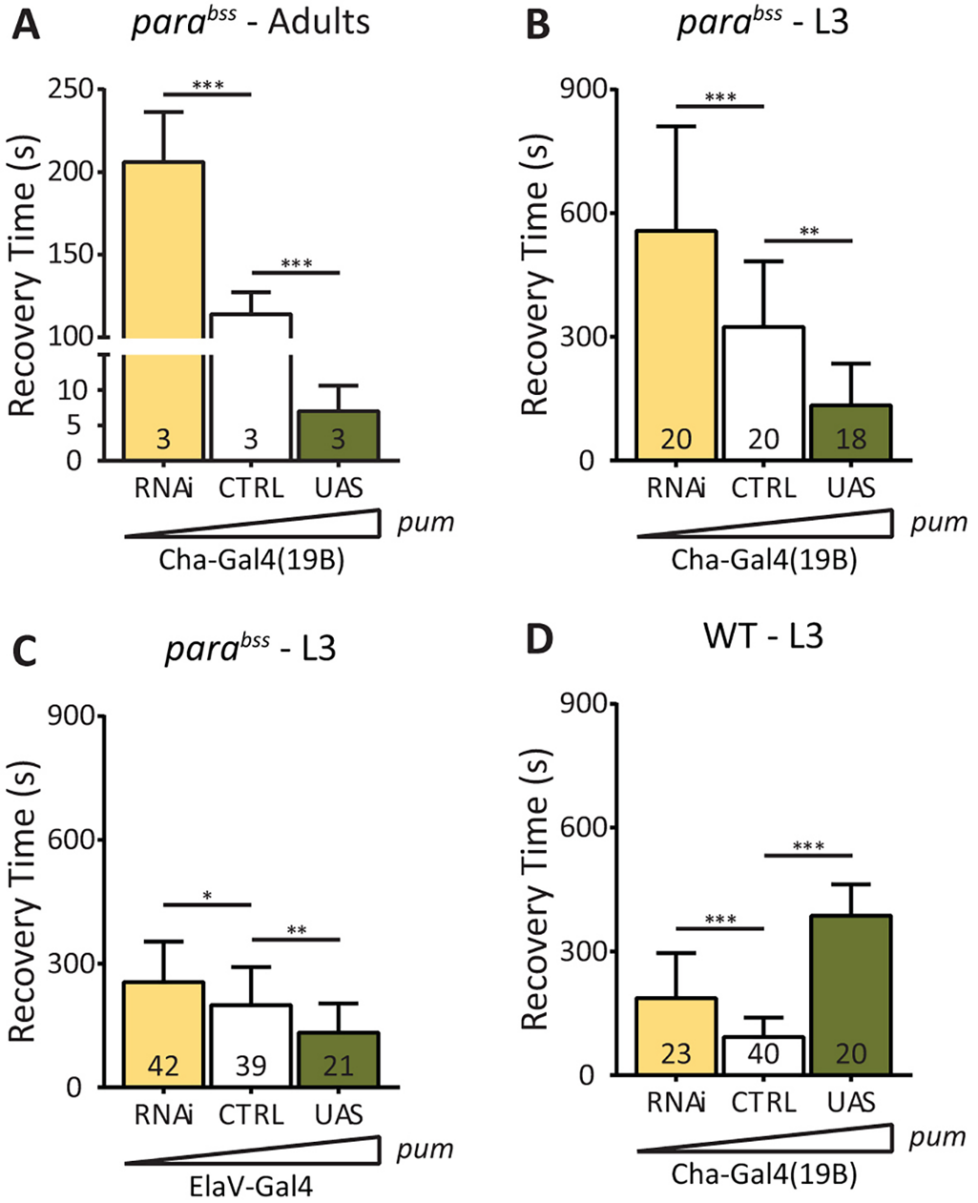
**Expression of transgenic *pum* is anticonvulsive.** (A) Expression of transgenic full-length *pum* lacking NRE motifs (UAS) in cholinergic neurons in *para^bss^* [Cha-Gal4(19B)>*pum*] is sufficient to reduce recovery time (RT) from mechanical shock-induced seizure in adult flies compared with *para^bss^* alone (CTRL). By contrast, further reduction of *pum*, through RNAi-mediated knockdown (RNAi) [Cha-Gal4(19B)>RNAi], significantly lengthens seizure RT. Each manipulation tested 10 flies per vial to produce an average value. This was repeated in triplicate and a final average calculated. Data are means±s.d., *n*=3. (B) Identical manipulation of *pum* expression in cholinergic neurons in L3 *para^bss^* had an identical effect on seizure duration when seizure was evoked by electroshock. (C) Pan-neuronal manipulation of *pum* is also sufficient to affect electroshock-induced seizure in L3. Upregulation (UAS: *para^bss^*;; elaV-Gal4>*pum*) reduces seizure and downregulation (RNAi: *para^bss^*;; elaV-Gal4>RNAi) increases seizure duration compared with control (*para^bss^*;; elaV-Gal4/+). (D) Up- or downregulation of *pum* in a WT background, using the Cha-Gal4(19B) cholinergic driver line, results in induction of a seizure phenotype. Data are means±s.d., *n* is stated in individual bars. *P≤0.05, **P≤0.01, ***P≤0.001 (two-way ANOVA with Bonferroni's post hoc).

We have shown that Pum binds to mRNA encoding Na_v_s in both *Drosophila* and rat. Binding subsequently reduces the density of Na_v_ channels available in the neuronal membrane (Driscoll et al., 2013; Mee et al., 2004; Muraro et al., 2008). We predicted, therefore, that maintaining *pum* expression in seizure backgrounds would be anticonvulsant. Inducing seizure by vortexing of *para^bss^*/Y male flies resulted in a recovery time (RT, 114±13.3 s, *n*=3) that was significantly reduced by exposure to recognised AEDs (Parker et al., 2011). Vortexing WT flies, by comparison, resulted in a near instantaneous RT (5.3±2.5 s, *n*=3). This is the averaged time taken for all flies in the vial (*n*=10) to regain a standing posture following vortexing and does not imply that WT flies exhibit seizures. By contrast, expressing *pum* in a Cha-Gal4(19B) cholinergic neuron driver line (which are the predominant excitatory interneuron type in the insect CNS; Yasuyama and Salvaterra, 1999), in *para^bss^* (*para^bss^*/Y; Cha-Gal4(19B)/UAS-*pum*) flies significantly reduced seizure RT compared with control *para^bss^*/Y; Cha-Gal4(19B)/+ (7±3.6 s vs 114±13.3 s, *P*=1.2×10^−5^, *n*=3; Fig. 2A). Indeed, recovery time following upregulation of *pum* was not significantly different to WT controls (5.3±2.5 s), indicative that seizures were completely suppressed. By contrast, expression of *pum*^RNAi^, using the same Cha-Gal4(19B) driver in the *para^bss^* background, was strongly proconvulsive (206±30.4 s vs 114±13.3 s, *P*=0.0002, *n*=3; Fig. 2A). We observed the same outcome in L3 where seizure behaviour was induced by electroshock (Fig. 2B). Seizure RT was significantly reduced (134±101 s, *P*=0.005, *n*=18) or increased (557±255 s, *P*=0.0004, *n*=20) by expression of either UAS-*pum* or UAS-*pum*^RNAi^, respectively, in Cha-Gal4(19B) cholinergic neurons in the *para^bss^* background (control *para^bss^*/Y; Cha-Gal4(19B)/+: 324±159 s, *n*=20). Manipulation of *pum* levels pan-neuronally (using *para^bss^*;; elaV-Gal4) resulted in an identical effect to electroshock-induced seizure in L3 (Fig. 2C). Increasing *pum* expression reduced seizure duration (133±70 s, *P*=0.009, *n*=21) and RNAi-mediated knockdown increased seizure duration (255±99 s, *P*=0.02, *n*=42) compared with control (204±88 s, *n*=39).

Manipulation of *pum* in a WT background resulted in a different outcome. Both RNAi-mediated knockdown and, particularly, overexpression of *pum* resulted in an induction of a seizure phenotype [Cha-Gal4(19B)/UAS-*pum*^RNAi^: 187±109 s, *P*=1.8×10^−5^, *n*=23; Gal4(19B)/UAS-*pum*: 387±77 s, *P*=2.8×10^−26^, *n*=20] compared with control [Cha-Gal4(19B)/+: 97±43, *n*=40; Fig. 2D]. This paradoxical result is similar to the effect of feeding WT *Drosophila* AEDs such as phenytoin that also result in seizure induction, an effect that has also been observed in rat (Callaghan and Schwark, 1980; Marley and Baines, 2011; Rundfeldt et al., 1990).

### Increased *pum* expression decreases I_NaP_ in motoneurons

Our previous work has shown that Pum regulates I_Na_ through translational regulation of *para* (Mee et al., 2004; Muraro et al., 2008). We recorded from *para^bss^*/Y L3 where the expression of transgenic *pum* was selectively manipulated in only the aCC motoneuron (using RRa-Gal4). Our choice to use this motoneuron is guided by the ability to combine genetics and electrophysiology; a selective Gal4 driver exists to express UAS-transgenes in this neuron, which is also accessible to patch electrodes. That I_NaP_ is greater in amplitude in aCC motoneurons in seizure mutants (Marley and Baines, 2011) is indicative that they share properties with central interneurons in human epilepsy, which can also show increased I_NaP_ (Stafstrom, 2007).

**Fig. 3.**
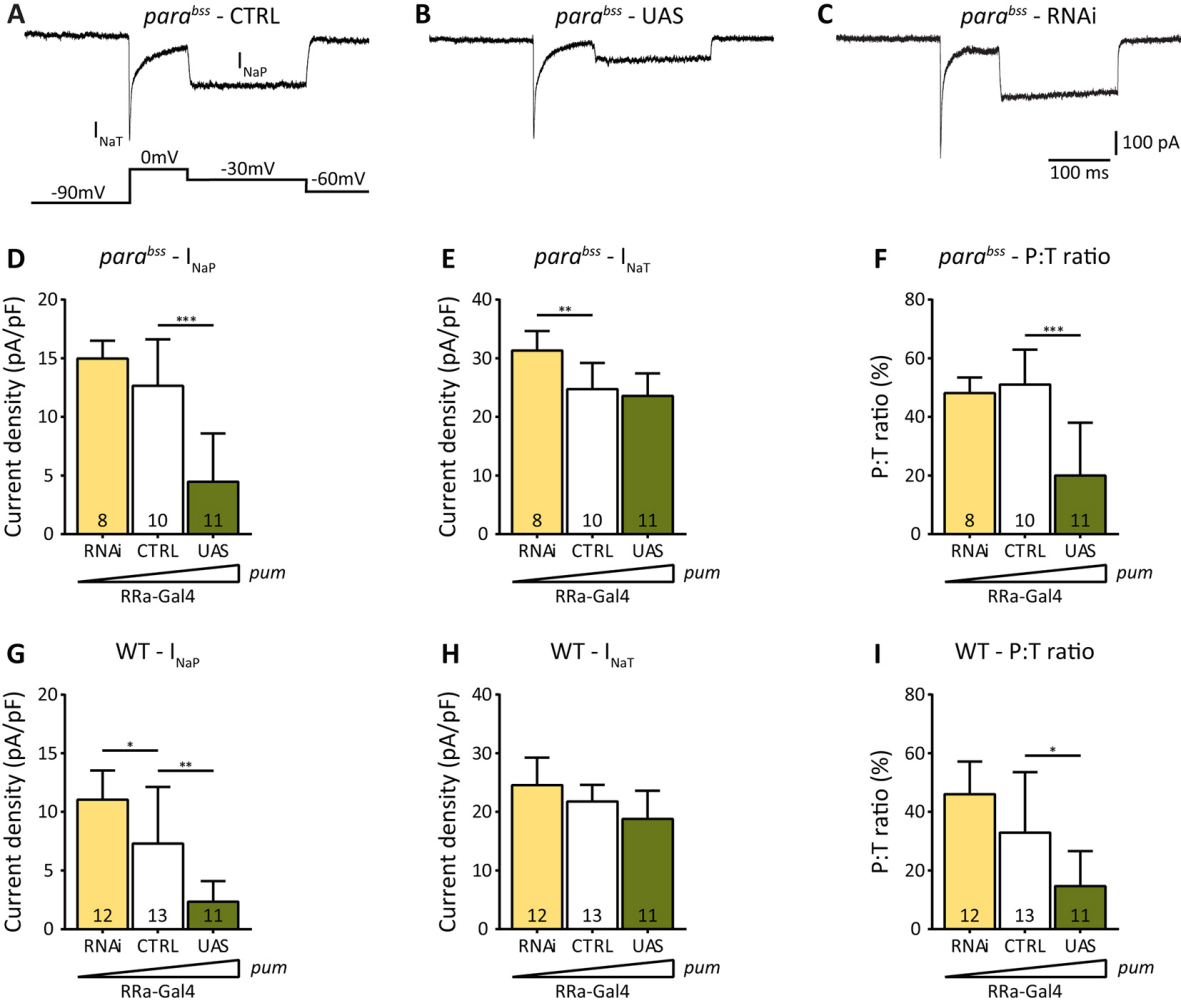
**Expression of transgenic *pum* reduces I_NaP_.** (A-C) Whole-cell patch recordings of I_Na_ from L3 aCC motoneurons in *para^bss^* (CTRL), *para^bss^* expressing transgenic *pum* (UAS) or *pum*^RNAi^ (RNAi). Transgene expression is limited to aCC motoneurons in these manipulations using RRa-Gal4. (D,E) Expression of transgenic *pum* (UAS) is sufficient to reduce the magnitude of I_NaP_ without change to I_NaT_. Expression of *pum*^RNAi^ (RNAi) results in no change to I_NaP_, but a significant increase in I_NaT_. (F) Persistent-to-transient (P:T) current ratio for I_Na_ recorded in D,E. (G,H) The effect of manipulating *pum* in a WT background. Increasing expression (UAS) is sufficient to reduce I_NaP_ with no change to I_NaT_, whereas reduction (RNAi) increases I_NaP_ amplitude but has no effect on I_NaT_. (I) Analysis of the P:T ratio in individual cells recorded in G,H shows increased *pum* is sufficient to reduce the ratio. Data are means±s.d. for *n* independent cells stated in individual bars. *P≤0.05, **P≤0.01, ***P≤0.001 (two-way ANOVA with Bonferroni's post hoc).

Increased expression of *pum* in L3 *para^bss^* aCC resulted in a striking reduction of I_NaP_ (4.4±4.1 pA/pF vs 12.6±4.0 pA/pF, *P*=4.9×10^−5^; Fig. 3A,B,D) but no change to I_NaT_ (Fig. 3E). Analysis of the persistent-to-transient current ratio (P:T) recorded in L3 aCC showed a marked reduction (20.0±18.0% vs 51.0±11.9%, *P*=5.0×10^−5^; Fig. 3F). A high P:T ratio (>40%) in central motoneurons has been previously shown to be characteristic of *Drosophila* seizure mutants and its reduction to be anticonvulsant (Lin et al., 2015; Marley and Baines, 2011). Thus, we conclude that upregulation of *pum* is anticonvulsant, which is due, at least partially, to its ability to reduce I_NaP_.

RNAi-mediated downregulation of *pum* in L3 *para^bss^* aCC increased I_NaT_ (31.3±3.3 pA/pF vs 24.7±4.5 pA/pF, *P*=0.005) but did not affect I_NaP_ or the P:T ratio (Fig. 3C-F). Analysis of the effect on seizure behaviour following this more selective manipulation of *pum* expression showed no significant differences to controls (*para^bss^*/Y;; RRa-Gal4/+, data not shown). This is entirely expected given the highly selective cell targeting used in these experiments. However, a more widespread manipulation of *pum* [e.g. using Cha-Gal4(19B)], which is sufficient to alter seizure duration and/or severity, probably acts via an identical mechanism: through alteration of I_Na_.

Increasing *pum* expression in aCC in a WT background resulted in essentially the same changes to I_Na_ as seen with manipulation in the *para^bs^*^s^ background; I_NaP_ was significantly reduced (2.4±1.7 pA/pF vs 7.4±4.9 pA/pF, *P*=0.0028; Fig. 3G) but no change to I_NaT_ was observed (18.8±4.8 pA/pF vs 21.9±2.7 pA/pF; Fig. 3H). By contrast, downregulation using *pum*^RNAi^ produced a different outcome compared with *para^bss^*; I_NaP_ was significantly increased (11.0±2.4 pA/pF vs 7.4±4.9 pA/pF, *P*=0.032; Fig. 3G) with no effect on I_NaT_ (24.6±4.7 pA/pF vs 21.9±2.7 pA/pF; Fig. 3H). Analysis of the P:T ratio, however, similarly only showed a significant reduction following upregulation of *pum* expression in WT (14.7±11.9% vs 33.3±20.2%, *P*=0.016; Fig. 3I).

**Fig. 4.**
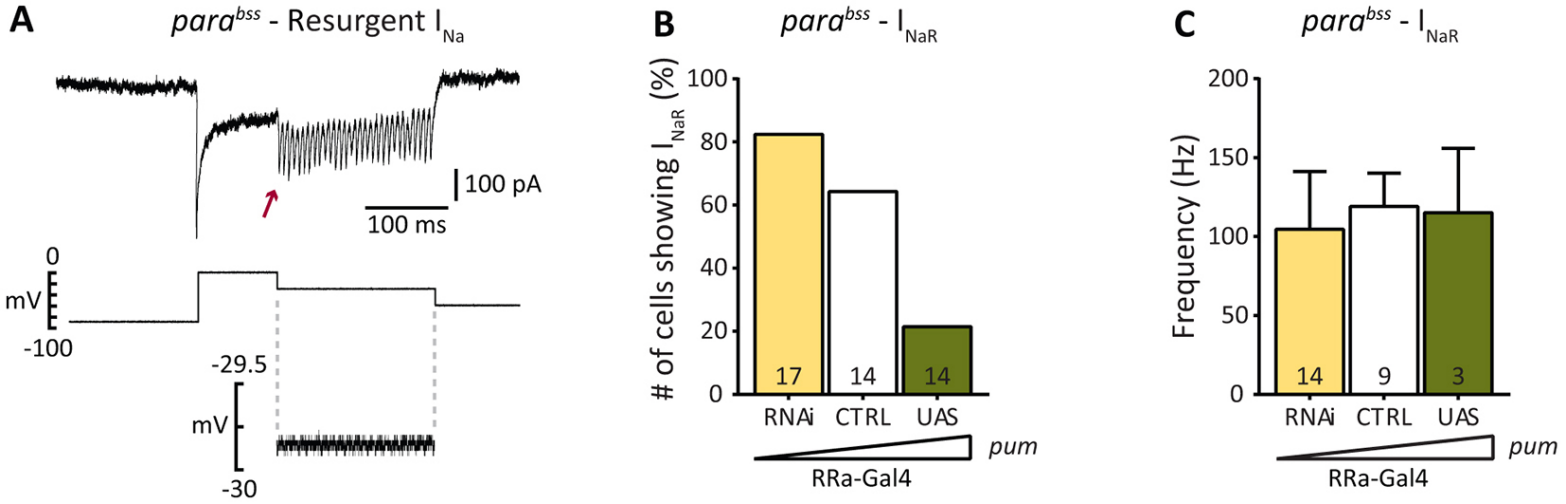
**Occurrence of resurgent I_Na_ is related to level of *pum*.** (A) Resurgent I_Na_ (I_NaR_, arrow) is seen superimposed on repolarization of holding potential used to evoke I_NaP_. Analysis of the voltage trace (lower trace) shows good control during this step. (B) The occurrence of I_NaR_, in the *para^bss^* background, is highest when *pum* is reduced (RNAi, 82%, 14 from 17 cells) and lowest when increased (UAS, 21%, 3 from 14 cells). Control (CTRL, *para^bss^*, 64%, 9 from 14 cells). Transgene expression was limited to aCC cells using RRa-Gal4. (C) Frequency of I_NaR_ oscillations is unaffected by expression level of *pum*. Data are means±s.d.

On occasion, we noted the appearance of multiple resurgent I_Na_ during the I_NaP_ plateau in the *para^bss^* background (Fig. 4A, indicated by arrow). Moreover, we observed a significant correlation between the occurrence of resurgent I_Na_ and *pum* level (*P*=0.002, Chi-square test; Fig. 4B). Thus, resurgent I_Na_ was most often observed following RNAi-knockdown, and only rarely following expression of *pum*. The origin of these currents remains uncertain. Analysis of voltage recordings (Fig. 4A) showed no obvious issue of space clamp, which suggests these currents are not occurring in distal unclamped regions of the neuron. The averaged frequency of the resurgent currents was ∼100 Hz, which did not vary with level of *pum* expression (Fig. 4C). Resurgent currents are particularly evident at holding potentials between −50 to −20 mV and exhibit highest frequency at −30 mV (RRa-Gal4/UAS-*pum*^RNAi^: 104.50±36.78 Hz; RRa-Gal4/+: 120.00±20.16 Hz; RRa-Gal4/UAS-*pum*: 115.00±40.93 Hz). Increased resurgent I_Na_ probably supports increased action potential firing consistent with our observation that RNAi-mediated knockdown of *pum* is proconvulsant (Grieco et al., 2005). Resurgent I_Na_ is only rarely observed (<5%) in WT aCC recordings (data not shown).

### A screen to identify positive regulators of Pum activity

Upregulation of Pum activity, either through increased transcription or post-transcriptional modification might provide an effective means to suppress seizures. To identify possible lead compounds with this mode of action, we constructed a luciferase-based reporter of Pum activity for use in an *in vitro* S2R+ cell line suited to large-scale screens (Lin et al., 2015). Overexpression of *pum* is sufficient to repress luciferase activity (due to translational repression), whereas incubation with *pum* double-stranded RNA is sufficient to increase luciferase activity by reducing endogenous Pum activity. PCR analysis shows that *pum* is endogenously expressed in S2R+ cells (Fig. S2). Thus, activity of the *firefly-luciferase-NRE* reporter (FF-NRE) reflects the absolute level of Pum function in these cells. A second reporter, which lacked an NRE-motif, was also transfected [*renilla* (*Ren*)-*luciferase*] to allow detrimental effects to cell viability to be determined. The final readout of the assay was a FF:Ren luciferase ratio that would be reduced following upregulation of Pum activity.

We screened 785 compounds from a repurposed library (see Materials and Methods; drugs screened are listed in Table S4). We identified 12 compounds that significantly reduced the FF:Ren ratio at 5 μM (Table 2). Based on structure and/or known drug target, the compounds fall into one of four groupings: those containing a methoxybenzaldehyde moiety (aniracteam and avobenzone); anti-cancer agents (cladribine, gemcitabine, floxuridine, clofarabine, bleomycin and docetaxel), mTOR inhibitors (temsirolimus and rapamycin) and topoisomerase II inhibitors (mitoxantrone and teniposide). Our attention was particularly drawn to avobenzone because, unlike the other compounds, it had no significant effect on transcription of the control *Ren-luciferase* reporter (all other compounds also reduced expression of this reporter, in addition to decreasing the FF:Ren ratio). Thus, we took avobenzone forward for further testing.

**Table 2.**
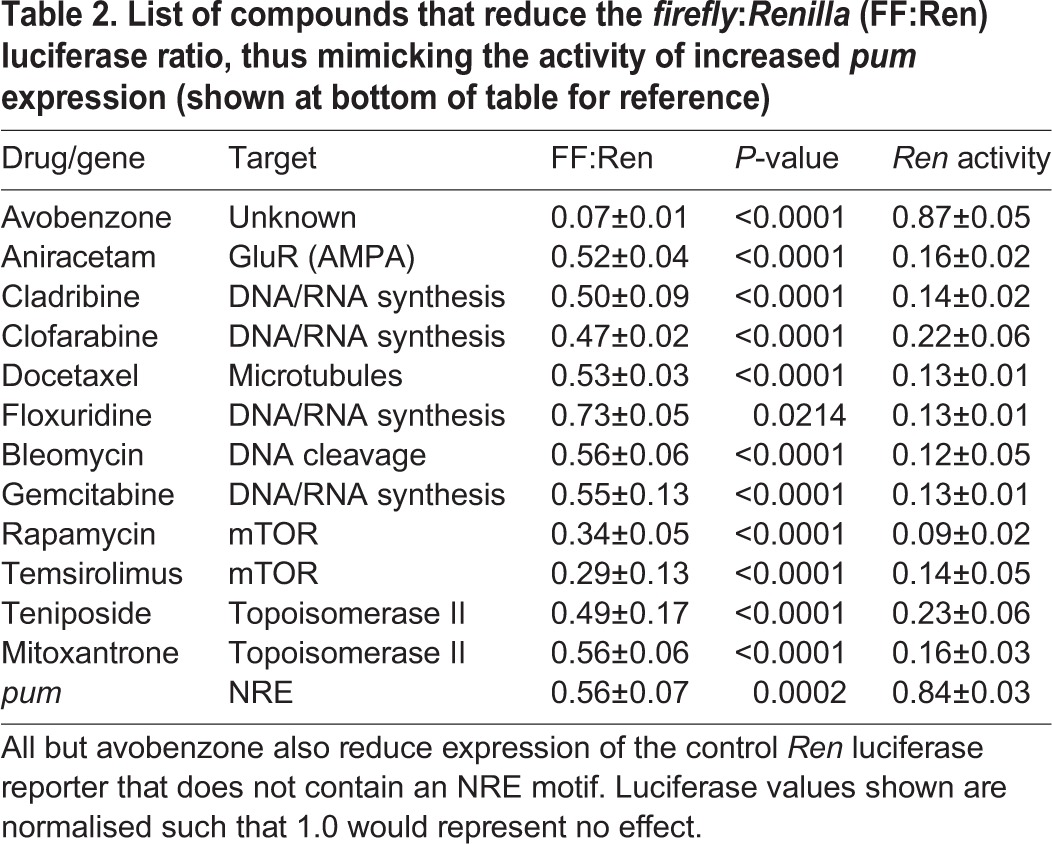
**List of compounds that reduce the *firefly*:*Renilla* (FF:Ren) luciferase ratio, thus mimicking the activity of increased *pum* expression (shown at bottom of table for reference)**

### Avobenzone potentiates activity of Pum

**Fig. 5.**
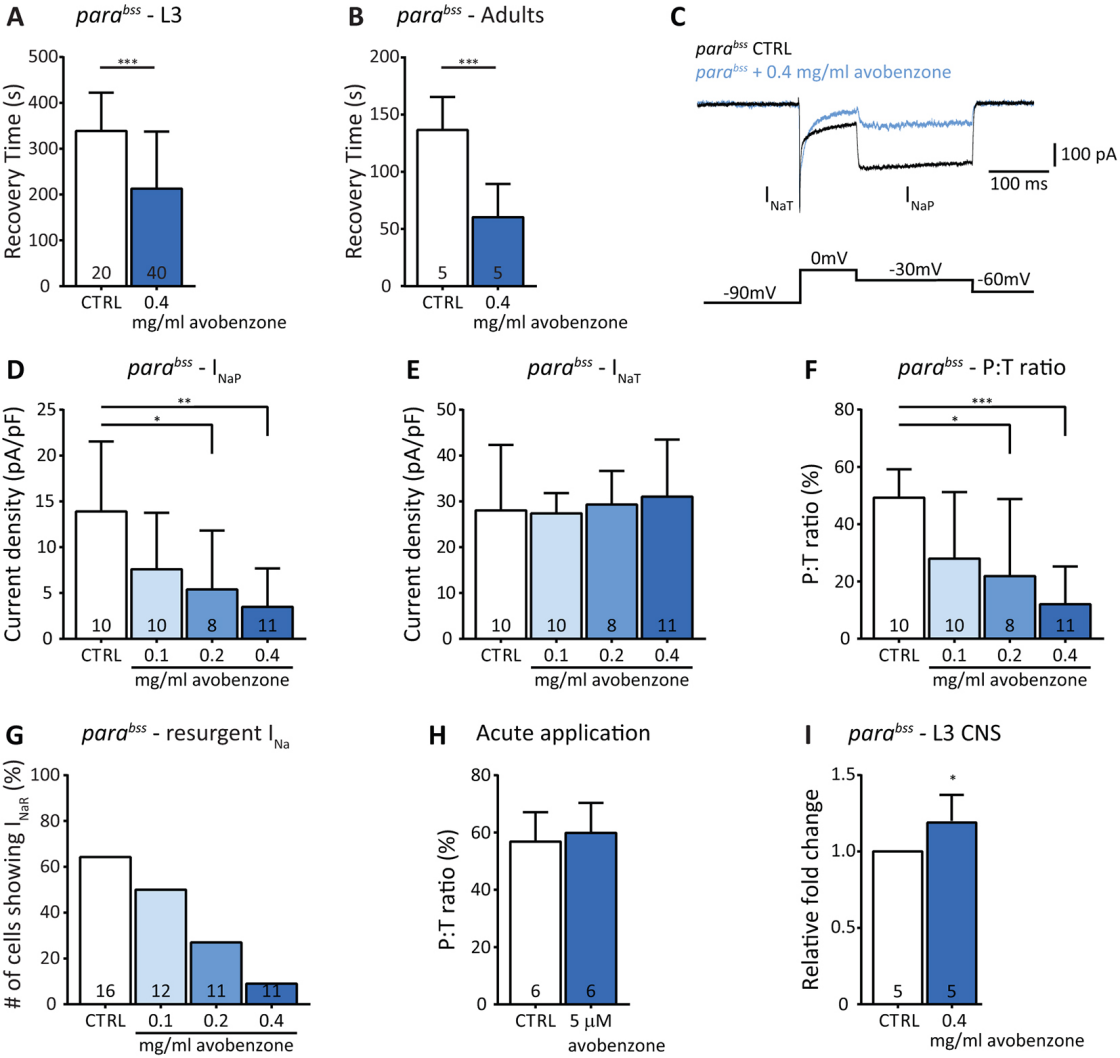
**Avobenzone is anticonvulsant and selectively reduces I_NaP_.** (A) *para^bss^* L3 raised in food containing 0.4 mg/ml avobenzone show significantly reduced recovery time (RT) following electroshock compared with controls (CTRL: *para^bss^*+DMSO). (B) Exposure of adult *para^bss^* flies to avobenzone (0.4 mg/ml) is also potently anticonvulsant compared with controls (CTRL: *para^bss^*+DMSO). Each manipulation tested 10 flies per vial to produce an average value. This was repeated five times and a final average calculated. (C) Whole-cell patch recordings of I_Na_ from *para^bss^* L3 aCC, raised in food containing 0.4 mg/ml avobenzone show reduced I_NaP_. (D,E) Increasing concentrations of avobenzone (0.1, 0.2 and 0.4 mg/ml) induced a proportional decrease of I_NaP_ (D) without affecting I_NaT_ (E). (F) Persistent-to-transient (P:T) current ratio for I_Na_ recorded in aCC. (G) The frequency of cells that exhibit resurgent I_Na_ correlates with avobenzone concentration (*P*=0.005, Chi-square test). (H) P:T ratio measured from *para^bss^* aCC before (CTRL) and after a 1 min bath application of 5 µM avobenzone. (I) Analysis of *pum* transcript level in isolated CNS from *para^bss^* L3 raised on food containing avobenzone (0.4 mg/ml) shows a significant increase compared with *para^bss^* L3 raised on food containing an equal amount of vehicle (0.8% DMSO). The control value has been set to 1. Data are means±s.d. for *n* independent cells stated in individual bars. *P≤0.05, **P≤0.01, ***P≤0.001 (A,H-I, unpaired *t*-test; D-F, two-way ANOVA with Bonferroni's post hoc).

We first tested for anticonvulsant activity in L3 *para^bss^* mutants. Larvae raised in food containing avobenzone (0.4 mg/ml) showed significantly reduced RT in response to electroshock (avobenzone: 213±124 s, *n*=40 vs control: 339±83 s, *n*=20, *P*=0.0004; Fig. 5A). Similarly, exposure of adult *para^bss^* flies to avobenzone (0.4 mg/ml), 24 h before testing, also resulted in significant reduction of seizure duration (avobenzone: 61±29 vs control: 138±29 s, *n*=5, *P*=0.0002, Fig. 5B). Next, we recorded I_Na_ from *para^bss^* aCC in L3 that had been raised on food containing different concentrations of avobenzone (0.1-0.4 mg/ml; Fig. 5C-G). Avobenzone reduced I_NaP_ from 13.9±7.6 pA/pF in controls to 7.6±6.2 pA/pF at 0.1 mg/ml (*P*=0.17), 5.4±6.4 pA/pF at 0.2 mg/ml (*P*=0.03) and 3.5±4.2 pA/pF at 0.4 mg/ml (*P*=0.002) (Fig. 5D). Conversely, avobenzone treatment at these concentrations did not induce any detectable effect in I_NaT_ (Fig. 5E). Analysis of the P:T ratio for I_Na_ shows that exposure to avobenzone significantly reduced this value from 49.3±9.2% in control to 28.0±23.2% at 0.1 mg/ml (*P*=0.09), 21.9±26.9% at 0.2 mg/ml (*P*=0.03) and 12.1±13.2% at 0.4 mg/ml (*P*=0.0004) (Fig. 5F), which compares favourably with overexpression of *pum* (c.f. Fig. 3). We also observed a significant correlation between avobenzone concentration and the occurrence of resurgent I_Na_ (*P*=0.005, Chi-square test, Fig. 5G).

Our predicted mode of action for avobenzone is inconsistent with an immediate effect of this compound, acting instead to potentiate Pum, which, in turn, downregulates Na_v_ channels in the neuronal membrane. To test this, we recorded from non-drug-exposed L3 *para^bss^* aCC and used bath application of avobenzone (5 µM). No changes were observed in either component of I_Na_ (data not shown) and the P:T ratio remained unaffected (Fig. 5H). Higher doses (20 µM), or longer exposure times (10 min) similarly produced no detectable effect (data not shown). This lack of acute effect is consistent with our predicted mode of action. Finally, to directly test this prediction, we measured *pum* transcript abundance in *para^bss^* L3 grown in the presence of avobenzone. We observed a modest, but statistically significant, increase in transcript abundance of ∼20% (1.2±0.17, *n*=5, *P*=0.04, *t*-test, vehicle control set as 1; Fig. 5I). Thus, we conclude that avobenzone, acting to increase the transcription and/or transcript stability of *pum*, is able to suppress seizure duration through downregulation of I_NaP_. Finally, we observed equally potent anticonvulsive activity of avobenzone in two other bang-sensitive mutants: *easily-shocked* (avobenzone: 142±82 vs control: 240±120 s, *n*=40, *P*=1.0×10^−5^, L3 electroshock) encoding an ethanolamine kinase (Pavlidis et al., 1994) and *slamdance* (avobenzone: 178±122 vs control: 272±108 s, *n*=40, *P*=6.8×10^−5^, L3 electroshock) encoding an aminopeptidase (Zhang et al., 2002), indicative that increasing Pum activity might be effective against a broad range of epilepsies.

## DISCUSSION

The causes of seizure, even in genetic epilepsies, vary greatly and are not confined to genes with obvious contributions to ion flux across neuronal membranes. This increases the challenge to identify individual mutations, to determine the physiological role of both the WT and mutated protein and, ultimately, to design drugs to minimise the unwanted effect of the mutation. In this study, we identify transcriptional changes that occur in the seizure-prone CNS. We identify over 700 common genes that show altered transcription in two different seizure models. It is noteworthy that we observed approximately double the number of genes showing altered transcription in *para^bss^* flies compared with those treated with PTX. The reason for this is unclear but might represent accumulated compensatory changes in the mutant line that have occurred in order to lessen the severity of seizure activity in *para^bss^* mutants. These additional genes warrant further investigation as potential seizure suppressors.

Many of the common transcriptional changes we identify, and in particular those that are upregulated (and thus open to inhibition by drug exposure), might provide effective drug targets for novel AED design. However, our attention was drawn to Pum, which we have previously shown orchestrates homeostasis of action potential firing in both *Drosophila* and rat central neurons (Driscoll et al., 2013; Mee et al., 2004). The degree of seizure suppression achieved by upregulating Pum in *para^bss^* flies is considerable and is only matched by the no-action-potential (nap^ts^) allele of the *maleless* (*mle*) locus in *Drosophila*, which encodes an ATP-dependent double-stranded RNA (dsRNA) helicase (Ganetzky and Wu, 1982). This mutation causes a catastrophic change in splicing of the *Drosophila* Na_v_ (Reenan et al., 2000). The net effect of both of these manipulations, increased Pum or the presence of *nap^ts^*, is to reduce the availability of functional Na_v_ expressed in central neurons. The direction of change of *pum* in the two seizure models (that show reduced expression) might not be ideal with respect to drug development given that disruption of a gene or protein is often more achievable. Nevertheless, we show that upregulation of *pum* in a *Drosophila* seizure mutant is potently anticonvulsive and, further, we identify a potential lead anticonvulsive compound that seemingly increases the level of expression of this homeostatic regulator. This compound might catalyse the development of a novel class of AED.

Neurons display an array of homeostatic mechanisms to maintain action potential firing within pre-determined and physiologically appropriate limits (Davis, 2013). Pum is a well-characterised RNA-binding protein that binds mRNA, usually through a specific motif termed the NRE. Once bound, Pum recruits additional cofactors including Nanos and Brain tumor (Brat) to form a complex that is sufficient to prevent translation (Wharton et al., 1998). Our results in this study indicate that increased expression of Pum might have therapeutic benefit for seizure suppression. However, a potential issue in this regard is that a genome-wide identification of RNAs bound to Pum in ovaries identifies upwards of 700 genes (FDR<0.1%) (Gerber et al., 2006). This raises the problem of specificity of effect following global potentiation of level or activity of Pum. This potential issue might, however, be overcome through identifying and targeting neuronal-specific regulators of Pum. One such alternative target might be the inhibition of Myocyte enhancer factor 2 (Mef2)-induced expression of miR-134 in neurons that, in turn, inhibits translation of mammalian PUM2 (Fiore et al., 2009). Additional possibilities include targeting of cofactors required for Pum activity. It is interesting in this regard that a loss-of-function mutation in mei-P26, a homologue of Brat, produces strong seizure suppression in *Drosophila* bang-sensitive seizure mutants (Glasscock et al., 2005).

Mammalian PUM2 binds transcripts encoding SCN1A (Na_v_1.1), and SCN8A (Na_v_1.6) (Driscoll et al., 2013; Vessey et al., 2010). A reduction in supply of Na_v_ protein to the neuron membrane is consistent with a reduction in action potential firing and a general anticonvulsant effect (Mee et al., 2004). Analysis of I_Na_ in motoneurons indicates that a likely mechanism includes a marked reduction in I_NaP_. Increased I_NaP_ is associated with mutations in *SCN1A* that have been identified from individuals with epilepsy (Meisler and Kearney, 2005) and is specifically reduced by AEDs such as phenytoin, valproate and lamotrigine (Stafstrom, 2007). In light of this, the anticonvulsant effect of increased *pum* expression is understandable. That reducing *pum* expression through RNAi-mediated knockdown is proconvulsive is again both predictable and understandable. However, the effect of this manipulation on I_Na_ is not so clear. Rather than increasing I_NaP_, I_NaT_ is instead significantly increased together with a novel appearance of resurgent I_Na_ during repolarisation. Increased I_NaT_ would be expected to reduce the threshold for action potential firing (i.e. making firing more likely), whereas resurgent I_Na_ is associated with increased action potential firing frequency, partly by reducing the refractory period (Grieco et al., 2005). Although we have observed this current component in recordings from seizure mutants (including *para^bss^*), it is rarely observed in WT or following expression of transgenic *pum*.

The ability to manipulate Pum *in vivo* to determine its anticonvulsive properties in rodent models of seizure will be greatly aided by the identification of chemical compounds that directly potentiate either expression or activity state. We report the use of a suitable cell-based screen to identify such compounds and highlight avobenzone as a potential lead compound for future development. The *in vivo* toxicity of avobenzone has not been well established and although there are few reports of serious side effects associated with its use as an active ingredient of sunscreen, its tendency to form free radicals might be a potential issue. To our knowledge, this compound has not been used to treat neurological disease, and its mode of action in reducing seizure in *Drosophila* remains to be determined. Our observations that ingestion of avobenzone result in increased expression of *pum* is indicative that this compound might mimic elements of the pathway that control expression of this homeostatic regulator.

The output of our screen also provides additional support for the use of rapamycin to control seizure (Lasarge and Danzer, 2014; Russo et al., 2013), indicative that this molecule might influence neuronal homeostasis. The identification of topoisomerase II as a potential target to control seizure also validates previous observations reporting that inhibition of this class of nuclear protein is anticonvulsant (Lin et al., 2015; Song et al., 2008). Finally, that we identify that the increase in Pum activity by aniracetam might hint at an additional mode of action for this class of known anticonvulsants (Shiotani et al., 2000). The related racetams, levetiracetam and brivaracetam, are currently in clinical use as AEDs, exploiting their capability to bind and inhibit synaptic vesicle protein 2A (SV2A) (Klitgaard et al., 2016).

In summary, we present a description of transcriptional change present in seizure-prone CNS. We identify, in particular, that *pum* expression is downregulated in both genetic and chemically induced seizure models. This mirrors the reported reduction in PUM2 in human TLE and in rats exposed to the proconvulsant pilocarpine (Wu et al., 2015). It also provides a possible understanding for why *Pum*2 null mice exhibit spontaneous seizures (Siemen et al., 2011). However, it is perplexing that *pum* levels should decrease during seizures given that the published model predicts an increase (Mee et al., 2004). As reduced Pum levels are predicted to increase neuronal excitability, it seems that epileptic seizures are associated with a pathological dysregulation of *pum* expression. We speculate that this occurs because Pum can auto-regulate (the *pum* transcript contains NRE motifs). Thus, although the neuronal hyperactivity induced by seizures will initially increase Pum expression, the accumulating Pum protein might feed back to downregulate its own transcript (Gerber et al., 2006). Sampling at later stages after seizure occurrence might only report reduced Pum compared with non-seizure controls. Indeed, we have shown that upregulation of *pum* in the *Drosophila* CNS, through expression of a wild-type transgene (lacking NRE motifs), results in reduction of endogenous *pum* transcript level (W.-H.L. and R.A.B., unpublished data). Prevention of this feedback, achievable in this study through expression of transgenic *pum* lacking an NRE, or exposure to avobenzone, holds significant promise for anticonvulsant therapy.

## MATERIALS AND METHODS

### Fly stocks

Wild type (WT, maintained in the Baines lab) was Canton-S. *para^bss^* (*bss^1^*), which was obtained from Dr Kevin O'Dell (Institute of Molecular, Cell and Systems Biology, University of Glasgow, UK), is detailed in Parker et al. (2011). The *para^bss^* stock (and other transgenic lines used) were not backcrossed to the CS stock. Controls consisted of either untreated *para^bss^* and/or parental stocks (i.e. Gal4/+, UAS/+) and are stated in respective figure legends. *Slamdance^iso7.8^* was obtained from Dr Mark Tanouye (Department of Environmental Science, Policy and Management and Department of Molecular and Cell Biology, University of California Berkeley, California, USA). *Easily-shocked^2F^* was obtained from Dr Kevin O'Dell. RRa-Gal4 is expressed in only the aCC and RP2 motoneurons (Lin et al., 2012). We are able to discriminate between these neurons during electrophysiological recordings and use only the aCC neuron in this study. We used Cha-Gal4(19B) to drive UAS-transgene expression in all cholinergic neurons, which include excitatory premotor interneurons (Salvaterra and Kitamoto, 2001). Pan-neuronal expression was achieved by combining elaV-Gal4 (Bloomington stock no. 8760, 3rd chromosome insert) with *para^bss^*. UAS-*pum*^RNAi^ was obtained from the Vienna *Drosophila* RNAi Center (stock no. 101399) and UAS-*pum* is detailed in Schweers et al. (2002). UAS-*pum* lacks NRE motifs that are present in the 3′-UTR of the endogenous *pum* gene. All genetic crosses were maintained at 25°C with the exception of overexpression of *pum* (larvae die as 1st or 2nd instars). These experiments were maintained at 20.5°C. Chemical-induced seizure was achieved by raising WT larvae on food containing 0.25 mg/ml PTX (P1675, Sigma, Poole, UK) until wall-climbing third instar, abbreviated to L3 (Lin et al., 2015).

### Library construction and RNA sequencing

CNSs were removed from 50 L3 (mixed sexes) and RNA extracted using the RNeasy mini kit (QIAGEN, Hilden, Germany) as described (Lin et al., 2015). RNA integrity and purity were determined using an Agilent 2200 TapeStation system (Agilent Technologies, Santa Clara, CA). The RNA-sequencing library was created using an mRNA Seq library preparation kit as per manufacturer's instructions (Illumina Inc., San Diego, CA). The library products were sequenced, in paired-end reads, using an Illumina HiSeqTM 2000. RNA-sequencing data were analysed using edgeR (empirical analysis of digital gene expression in R) (Robinson et al., 2010). This analysis identified genes with altered levels of expression using a threshold false discovery rate (FDR)≤1%. GO terms for Biological Process, Cellular Component, Molecular Function and Kyoto Encyclopedia of Genes and Genomes (KEGG) pathway were used for annotations. We classified differentially expressed genes using the Functional Annotation Cluster (FAC) tool available in the Database for Annotation, Visualization and Integrated Discovery (DAVID) (Huang et al., 2009a,b).

### Validation of RNA-sequencing analysis by quantitative PCR

Quantitative RT-PCR was performed using a SYBR Green I real-time PCR method (Roche, LightCycler^®^ 480 SYBR Green I Master, Mannheim, Germany) as described in Lin et al. (2015). RNA was extracted from either 20 adult heads (3 days old) or 20 L3 CNSs (mixed sexes) using the RNeasy micro kit (QIAGEN). Primer sequences (5′ to 3′) used were: *actin-5C* (CG4027), CTTCTACAATGAGCTGCGT and GAGAGCACAGCCTGGAT; *pum* (CG9755), GCAGCAGGGTGCCGAGAATC and CGCGGCGACCCGTCAACG (forward and reverse, respectively). Relative gene expression was calculated as the 2^−ΔCt^, where ΔCt was determined by subtracting the average *actin-5C* Ct value from that of *pum*.

### Luciferase reporter construction

A region of the 3′UTR (NM_169233.2, 2390-2650) of *hunchback*, containing two *pum*-binding motifs (NRE^1^ and NRE^2^) (Gupta et al., 2009), was subcloned from UAS-*firefly*-NRE/pUAST (a gift from Dr Kevin Moffat, University of Warwick, UK) by releasing the DNA fragment using *Eco*RI and *Xho*I sites and ligating it into pAc5.1 vector (Invitrogen). *Renilla* luciferase was subcloned from pRL-CMV vector (Promega) by releasing the DNA fragment using *Nhe*I (filling the sticky end to blunt end with Klenow) and *Xba*I sites and ligating it into *Eco*RV and *Xba*I sites of pAc5.1 vector (Invitrogen).

### Compound library screen

S2R+ cells (1.5×10^4^ cells in 15 µl of Schneider's *Drosophila* Medium, Gibco) were treated with 5 µl drug (final concentration 5 µM with 0.5% DMSO) in 384-well plates (Selleckchem) for 48 h, followed by co-transfection (Effectene, QIAGEN) of *firefly*-NRE and *renilla* luciferase reporters (10 ng each) for a further 48 h. The transfection procedure is as described in the manufacturer's instructions (QIAGEN). S2R+ cells were lysed with 0.35% Triton X-100 in BL buffer (50 mM HEPES, 0.5 mM EDTA, 0.36 mM phenylacetic acid and 0.07 mM oxalic acid), and D-Luciferin (0.46 mM, Molecular Probes) was added to measure *firefly* luciferase activity. This was followed by adding coelenterazine-h (3 mM, Promega) to measure *renilla* luciferase activity. A Varioskan flash plate reader (Thermo Scientific) was used to measure luminescence.

### Seizure behaviour test

Twenty virgin females of *para^bss^*; Cha-Gal4(19B) were mated with five males of UAS-*pum*^RNAi^, UAS-*pum* or WT. Because *para^bss^* is on the X chromosome and heterozygous *para^bss^*/+ females show significantly reduced recovery time, we used *para^bss^*/Y male F1 progeny for behavioural screening. For adult seizure determination, male flies (3 days old) were tested at least one day after collection to ensure total recovery from CO_2_-anaesthesia. Ten flies were transferred to an empty plastic fly vial and left to recover for 30 min before a mechanical shock induced by vortexing the vial at maximum speed for 10 s. Recovery time (RT) was calculated from the average time taken for all 10 flies to recover from paralysis to standing (to produce a single value). At least three replicates (of 10 flies per vial) were performed for each condition tested and the recovery time averaged across the three vials. Avobenzone was fed to young adult male flies (*para^bss^*/Y), within 8 h of eclosion. Groups of 10 flies were placed in an empty vial and exposed to drug-soaked filter paper. Drug was first mixed with a sucrose solution (5%) to produce a final concentration of 0.4 mg/ml (1.6% DMSO). Filter paper soaked in this solution was added to vials and left for 24 h before testing.

To measure seizure in larvae, an electroshock assay was performed as previously described (Marley and Baines, 2011). Briefly, L3 male larvae (*para^bss^*/Y) were transferred to a plastic dish after washing to remove food residue and gently dried using paper tissue. Once normal crawling behaviour resumed, a conductive probe composed of two tungsten wires (0.1 mm diameter, ∼1-2 mm apart) was positioned over the approximate position of the CNS, on the anterior-dorsal cuticle of the animal. A 30 V DC pulse for 3 s, generated by a Grass S88 stimulator (Grass instruments, RI, USA) was applied. In response to the electric stimulus, we observed a transitory paralysis in which larvae tonically contracted and, occasionally, exhibited spasms. The time to resumption of normal crawling behaviour was measured as RT. For drug-feeding studies, larvae were raised on food containing avobenzone (PHR1073, Sigma), in 0.8% DMSO, until reaching L3.

### Electrophysiology

Whole-cell voltage-clamp recordings were performed on aCC motoneurons at L3 as previously described (Marley and Baines, 2011). Leak currents were subtracted on-line (P/4). The same stimulation protocol was applied three times to each neuron and the recordings averaged. Current amplitudes were normalised for cell capacitance, determined by integrating the area (1 ms time range) under the capacity transients elicited by stepping the cell from −60 to −90 mV for 30 ms. Cells exhibiting no measurable I_NaP_ (resulting from excessive resurgent I_Na_) were not included in the quantitative analysis.

To evaluate the effect of *pum* manipulation on I_Na_, virgin females of *para^bss^*;; RRa-Gal4 were crossed with UAS-*pum*^RNAi^, UAS-*pum* or WT males. Only *para^bss^*/Y male F1 progeny was recorded at L3. To investigate avobenzone action, *para^bss^*;; RRa-Gal4 larvae were raised on food containing 0.8% DMSO or avobenzone at different concentrations (0.1, 0.2 and 0.4 mg/ml) until reaching L3. Acute drug treatment was performed by bath-applying avobenzone to the external saline (0.5% DMSO). I_Na_ was recorded from *para^bss^*;; RRa-Gal4 aCC motoneurons before and 1 min after bath application. Controls were exposed to DMSO alone.

### Statistics

Statistical significance between group means was assessed using either a Student's *t*-test (where a single experimental group is compared with a single control group) or ANOVA followed by the Bonferroni's post hoc test (multiple experimental groups). The Chi-square test was used for statistical analysis of categorized data. Data shown is mean±s.d.
